# Ontogeny of zebrafish behaviors: comparative evaluation of locomotor, social and anxiety parameters in larval, juvenile and adult stages

**DOI:** 10.1038/s41684-026-01712-x

**Published:** 2026-04-07

**Authors:** Barbara D. Petersen, Gabriel Rodrigues, Kirya Liriel, Lana Ferreira, Carla D. Bonan

**Affiliations:** 1 Programa de Pós-Graduação em Medicina e Ciências da Saúde, Escola de Medicina, Pontifícia Universidade Católica do Rio Grande do Sul, Porto Alegre, Brazil; 2 Laboratório de Neuroquímica e Psicofarmacologia, Escola de Ciências da Saúde e da Vida, Porto Alegre, Brazil; 3 Programa de Pós-Graduação em Biologia Celular e Molecular, Escola de Ciências da Saúde e da Vida, Pontifícia Universidade Católica do Rio Grande do Sul, Porto Alegre, Brazil

**Keywords:** Development of the nervous system, Zebrafish

## Abstract

Zebrafish are a prominent model for investigating behavior and development. However, most behavioral studies have primarily focused on larval and adult stages. The juvenile stage—a critical period of neural and behavioral maturation—has been insufficiently explored, partly because standardized methods for evaluating behavior in different ages are not available. Here we present a behavioral platform adapted for cross-stage assessments and provide an initial characterization of juvenile zebrafish behavior. Locomotion, anxiety, social interaction and scototaxis were evaluated. Our findings reveal developmental stage-specific differences in juvenile zebrafish locomotion, such as increased mobility and reduced erratic movements, along with a steady and progressive increase in social preference from late larval to the adult stage. Scototaxis reversal was also found to be one of the first major behavior transitions, occurring early in the larval stage. These results establish juveniles as a transitional phase with distinct phenotypes and support previous hypotheses that a behavioral metamorphosis accompanies morphological changes of juveniles in these species. Furthermore, this study establishes a foundation for longitudinal behavioral analyses, with the standardization of tests enabling further studies on neurodevelopmental disorders, pharmacological interventions and behavioral ontogeny.

## Abstract (web summary)

This study introduces a platform for evaluating zebrafish behavior across developmental phases. The results show juveniles as a transitional and critical developmental phase and establish a foundation for longitudinal behavioral analyses.

## Main

Zebrafish are a vertebrate model species, disseminated in laboratories from the 1970s onward^1^ and extensively used in a variety of research fields, such as genetics, pharmacology, developmental biology and neuroscience^2–5^. As an animal model, zebrafish are an excellent alternative for early developmental studies due to their short embryogenesis^6^, the ability to evaluate behavior as early as 17 h post-fertilization^7^, their capacity to absorb water-soluble compounds^8,9^ and transparent embryos that allow in vivo phenotype observations^10^. Adult zebrafish maintain some of these characteristics, such as the possibility to expose them to compounds directly in the tank water, while also displaying a variety of behavioral phenotypes^11^ and functional homology in neuronal circuits compared to mammals^12,13^. By contrast, other developmental stages of zebrafish, such as the juvenile and elderly stages, remain underrepresented and understudied in the current literature.

The juvenile stage, the primary focus of our investigation, corresponds to adolescence in mammals^14,15^. This is a sensitive period, marked by extensive physical, physiological, cerebral and behavioral changes^16^. When atypical processes occur during the changes in neural systems underlying cognitive function, social interaction, emotional control, risk–reward balance and motivation during adolescence, psychiatric conditions such as depression, anxiety disorders and psychosis may emerge or worsen. This is particularly relevant when these abnormal changes are associated with an increased propensity of risk-taking, novelty seeking and other behaviors typical of this developmental stage^16–21^. Nevertheless, the relationship between the changes that occur during adolescence and the development of psychopathologies is complex and not linear. In addition, methodological limitations in studying this issue in mammals remain a substantial challenge^16^. In this scenario, modeling adolescence in alternative animal models, such as zebrafish, can be useful not only for testing new interventions and unraveling disease mechanisms^22,23^, but also because comparative studies between species can reveal evolutionary conserved functions, mechanisms and targets, thus facilitating the elucidation of central aspects of diseases^24,25^.

The juvenile stage of zebrafish’s life cycle is poorly standardized in literature. First, animals classified as juveniles can be found in the literature ranging from as early as 6 days post-fertilization (dpf) to as late as 70 dpf (refs. ^26,27^). Other studies suggest the juvenile stage encompasses the period between ~30 dpf and ~90 dpf (refs. ^14,28^). These discrepancies can be partly explained by the influence of factors, such as temperature, population density, stress levels and others, which can affect developmental rates in this species and delay metamorphosis^14^. This has led to the proposal of using ecological and anatomical hallmarks as more reliable criteria for determining life stages, rather than relying only on dpf^14,29,30^. Second, innate behaviors, which directly affect animal survival and serve as a functional reading of neural activity^31–33^, show noticeable differences between larval and adult zebrafish; however, the timeline of when exactly these changes occur has not yet been elucidated. In this context, the transition from the larval to the juvenile stage has been proposed to involve a behavioral metamorphosis, marked by enhanced reactivity to stimuli^34^. This hypothesis remains untested, partly because the current methods for evaluating behaviors in zebrafish are not standardized to allow comparisons between life stages^35^.

Therefore, this study aims to develop a behavioral analysis platform that allows direct comparisons between the early larval, late larval, early juvenile, late juvenile and adult life stages of the zebrafish model as an initial effort for characterizing behaviors of juvenile zebrafish, focusing on locomotion and social interaction.

## Results

### Novel tank test

The anatomical parameters for determining life stages are described in the Table 1.

**Table 1:** Anatomical parameters for determining life stages

Developmental stage	Description
Early larval stage, 4.5 mm, 7 dpf	•Absence of defined rays in the fins
	•Posterior lobe of the swim bladder inflated
Late larval stage, 7.6 mm, 21 dpf	•Defined rays and incomplete pigmentation in the anal, caudal and dorsal fins
	•Anterior lobe of the swim bladder inflated
	•Absence of defined rays in the pelvic fin
Early juvenile stage, 9.8 mm, ~30 dpf	•Pelvic fin with defined rays and incomplete coloration
	•Incomplete resorption of the fold between the pelvic fin and cloaca
	•Incomplete scaling
Late juvenile stage, 13 mm, ~45 dpf	•Complete pigmentation of the pelvic fin
	•Absence of fold between the pelvic fin and cloaca (complete resorption)
	•Complete scaling
Adult stage, 26 mm, >90 dpf	•Animals with complete sexual dimorphism
	•Male: slim body, pinkish coloration
	•Female: bulging body in the ventral region, silvery coloration

**Fig. 1:**
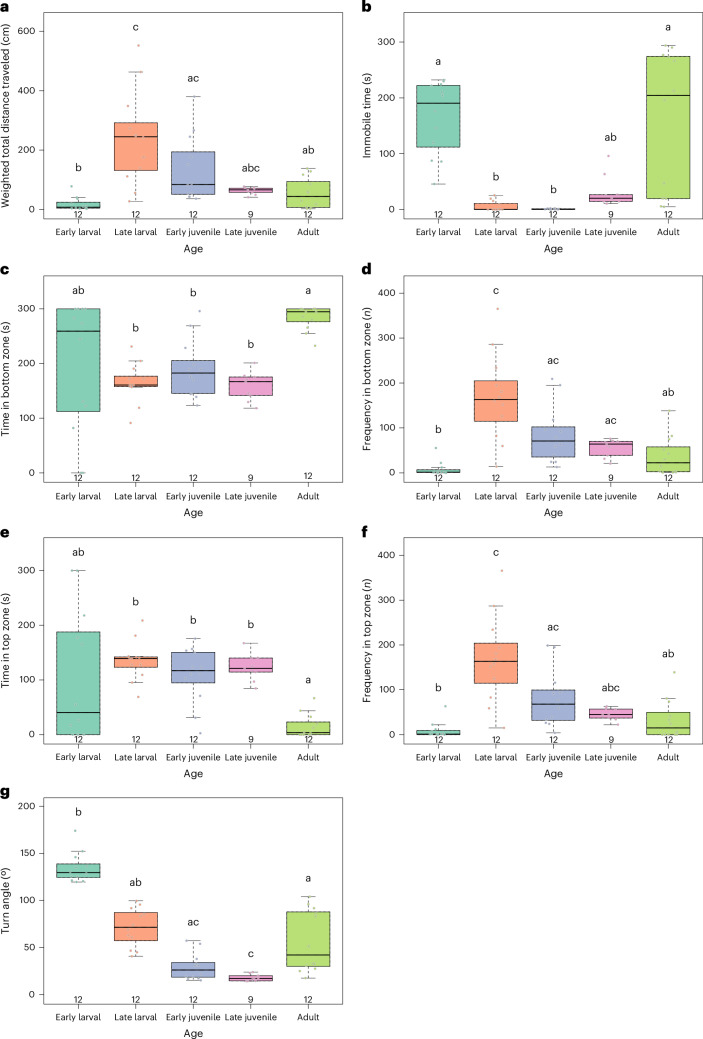
Locomotor behavior of zebrafish in the novel tank test across life stages. **a**, Weighted distance traveled (cm). **b**, Immobile time (s). **c**, Time in the bottom zone (s). **d**, Frequency in the bottom tank zone (*n*). **e**, Time in the top tank zone (s). **f**, Frequency in the top tank zone (*n*)). **g**, Turn angle (°). Animals from both sexes were analyzed at distinct developmental stages: Early larval (7 dpf), mid-larval (14 dpf), late larval (21 dpf), early juvenile (~30 dpf), late juvenile (~45 dpf) and adult (>90 dpf). Anatomical characteristics pertaining to age and sex are detailed in Table 1. Data were analyzed using the Kruskal–Wallis test followed by Dunn’s post hoc test and are expressed as median ± IQR. Sample sizes are shown in the figure. Different letters indicate statistically different groups. Detailed statistical values are provided in ‘Results’ and Supplementary Table 1.

To analyze baseline (naive animals) locomotor changes between ages, we first analyzed the total time spent in the novel tank test, comparing each life stage with all the other stages using the Kruskal–Wallis test with Dunn post hoc test. Our results showed significant differences between life stages for weighted distance traveled (*H* = 28.0566; *P* < 0.0001), bottom frequency (*H* = 32.0183; *P* < 0.0001), bottom time (*H* = 19.9972; *P* = 0.0005), top frequency (*H* = 29.9885; *P* < 0.0001), top time (*H* = 20.2141; *P* = 0.0004), immobile time (*H* = 40.2210; *P* < 0.0001) and turn angle (*H* = 44.3140; *P* < 0.0001) (Fig. 1).

For the weighted total distance traveled parameter **(**Fig. 1a), animals in the late larval stage traveled a greater distance than animal in the early larval (*P*_Dunn_ < 0.0001) and adult (*P*_Dunn_ = 0.0103) stages. Early larval fish also traveled less than early juvenile fish (*P*_Dunn_ = 0.0030). This may be due to the high immobile time (Fig. 1b) displayed by early larval fish in comparison with late larval (*P*_Dunn_ < 0.0001) and early juvenile fish (*P*_Dunn_ < 0.0001). Adult fish also showed increased immobile time in comparison with late larval (*P*_Dunn _= 0.0007) and early juvenile fish (*P*_Dunn_ < 0.0001).

Regarding tank exploration, animals showed different patterns depending on development. While animals in the intermediate stages (late larval to late juvenile) tended to explore the entirety of the tank and shift constantly between zones, early larval fish shifted zones less often; however, time in each zone was not significantly different from the other groups. By contrast, adult fish, which also changed zone less frequently, showed a preference for the bottom zone of the tank in comparison with fish in all other stages.

This can be seen by analyzing the top (Fig. 1f) and bottom frequencies (Fig. 1d) and top (Fig. 1e) and bottom (Fig. 1c) times. Early larval animals entered the top zone significantly less than their late larval (*P*_Dunn_ < 0.0001) and early juvenile (*P*_Dunn_ = 0.0087) counterparts. Bottom frequency was also decreased for early larvae compared with late larval (*P*_Dunn_ < 0.000), early juvenile (*P*_Dunn_ < 0.0001) and late juvenile (*P*_Dunn_ = 0.04933) stages. As stated above, no differences were found in the time spent in either the bottom or top zone for early larval fish compared with fish in all other stages. In adults, however, the frequencies to enter both the top (*P*_Dunn_ = 0.0009) and bottom (*P*_Dunn_ = 0.0036) zones were reduced compared with late larval fish, the stage showing the higher frequency of transitions; this can be explained by the increase in time spent in bottom zone by adult fish compared with late larval (*P*_Dunn_ = 0.0015), early juvenile (*P*_Dunn_ = 0.0238) and late juvenile (*P*_Dunn_ = 0.0017) and reduced time spent in the top zone compared with late larval (*P*_Dunn_ = 0.0003), early juvenile (*P*_Dunn_ = 0.0119) and late juvenile (*P*_Dunn_ = 0.0113) fish.

At first, we hypothesized that this increase in shifts between zones for intermediate life stages could be caused by a more erratic swim pattern. However, the turn angle (Fig. 1g) parameter, which indicates angular changes in direction, showed a gradual decline of amplitude of angles from the early larval stage to the late juvenile stage, followed by an increase in the adult stage. In this context, early larval animals showed the highest turn angle, differing significantly from early juvenile (*P*_Dunn_ < 0.0001), late juvenile (*P*_Dunn_ < 0.0001) and adult fish (*P*_Dunn_ = 0.0068), while late juvenile fish had the lowest turn angle, which was significantly lower than early larval (*P*_Dunn _< 0.0001), late larval (*P*_Dunn_ = 0.0022) and adults (*P*_Dunn_ = 0.0463).

To further investigate the behaviors across developmental stages in the novel tank test, we investigated the interactions of age and elapsed time by extracting the novel tank test results in 1-min intervals from the Ethovision software.

### Novel tank results per 1-min intervals

**Fig. 2:**
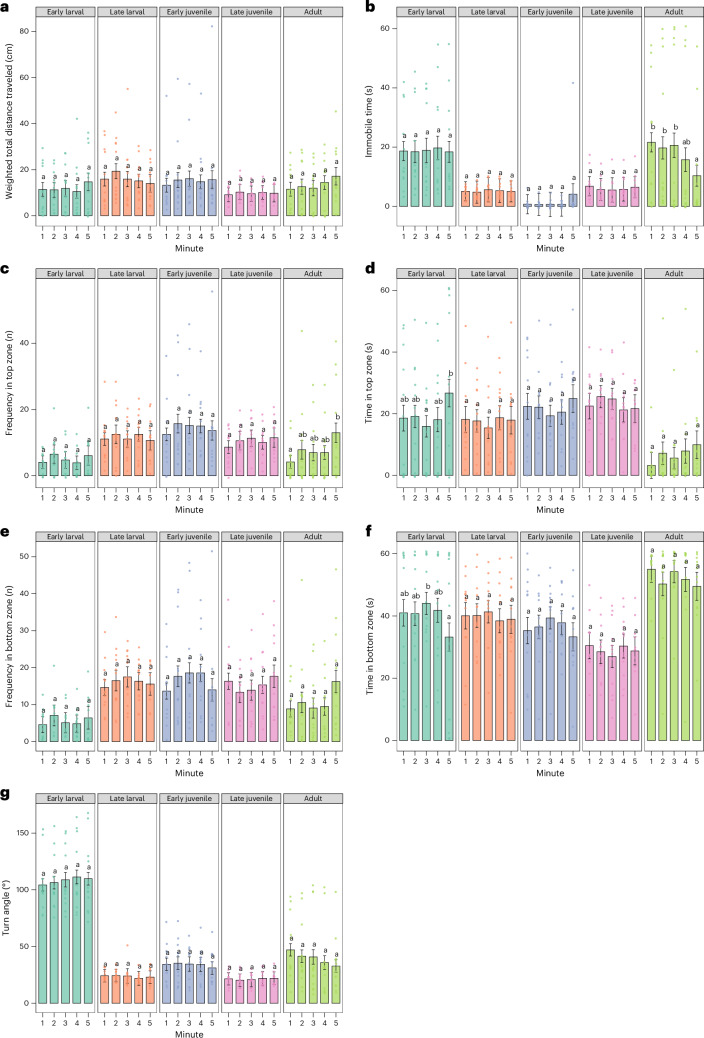
Per-minute intervals of zebrafish behavior in the novel tank test across life stages. **a**, Weighted distance traveled (cm). **b**, Immobile time (s). **c**, Frequency in the top tank zone (*n*). **d**, Time in the top tank zone (s). **e**, Frequency in the bottom tank zone (*n*). **f**, Time in the bottom tank zone (s). **g**, Turn angle (°). Animals from both sexes were analyzed at distinct developmental stages: Early larval (7 dpf), mid-larval (14 dpf), late larval (21 dpf), early juvenile (~30 dpf), late juvenile (~45 dpf) and adult (>90 dpf). Anatomical characteristics pertaining to age and sex are detailed in Table 1. Data were analyzed by RM-ANOVA followed by Tukey’s post hoc test and are expressed in mean ± s.d. Sample sizes are shown in the figure. Different letters show significant differences between minutes within each age. Detailed statistical values are provided in ‘Results’ and Supplementary Table 1.

Analysis of the novel tank test by minute intervals was conducted by two-way repeated measures analysis of variance (RM-ANOVA) with ‘age’ as the between-subjects factor and ‘minute’ as the within-subjects (repeated measures) factor, followed by a Tukey post hoc test. When analyzing the data across intervals, we found no differences in the weighted total distance parameter, either for the minute of the test as a variable or for the interaction between age and minute of the test (Fig. 2a, Supplementary Fig. 1a and Supplementary Table 1). Of note, some trends were observed for minute as a factor for bottom time (*F*_(4,54)_ = 2.5934, *P* = 0.0556), top frequency (*F*_(4,54)_ = 2.4989, *P* = 0.06379) and top time (*F*_(4,54)_ = 2.2604, *P* = 0.0846). The main finding, however, is that age was the main factor responsible for the differences observed in immobile time (*F*_(4,54)_ = 5.6928, *P* = 0.0006), top frequency (*F*_(4,54)_ = 2.8145, *P* = 0.0340), top time (*F*_(4,54)_ = 4.0069, *P* = 0.0064), bottom frequency (*F*_(4,54)_ = 4.3864, *P* = 0.0038), bottom time (*F*_(4,54)_ = 6.2969, *P* = 0.0003) and turn angle (*F*_(4,54)_ = 2.8145, *P* = 0.0340).

For immobile time (Fig. 2b and Supplementary Fig. 1b), all stages, except the adult stage, showed a uniform value throughout the test (Fig. 2b). Adult fish showed a decrease in immobility from the 4th minute forward, with the 5th minute of test displaying the lower immobility and differing significantly from minutes 1 (*P*_Tukey_ = 0.0015), 2 (*P*_Tukey_ = 0.0124) and 3 (*P*_Tukey_ = 0.02279). When comparing stages at each test minute, early larval fish were less mobile than late larval and early juvenile fish throughout the test, but especially in minutes 1, 2 and 5 (Supplementary Table 1). Adult fish were also less mobile than late larval and juvenile fish in the first minutes of testing (Supplementary Table 1).

Similarly, for the frequency in the top zone (Fig. 2c and Supplementary Fig. 1c) we observed intratest variation only in the adult stage, between the 1st and 5th minute of test (Fig. 2c, *P*_Tukey_ = 0.0104). In addition, early larval animals entered the top zone less frequently than early juveniles in minutes 1 (*P*_Tukey _= 0.0421), 3 (*P*_Tukey_ = 0.0431) and 4 (*P*_Tukey_ = 0.0039) and less frequently than late larval fish in minute 4 (*P*_Tukey_ = 0.0040) (Supplementary Fig. 1c).

In early larval zebrafish, time in the top zone (Fig. 2d and Supplementary Fig. 1d) increased in the last minute of testing, in comparison with the 3rd (*P*_Tukey_ = 0.0064) and 4th (*P*_Tukey_ = 0.0181) minutes, significantly (Fig. 2d). Although we anticipated an increase in top time in the last minutes of testing for the adult stage, our data do not reflect this expected pattern for this stage (Fig. 2d). However, when comparing adults with the other stages, we found significant differences throughout the entirety of the task, specifically in minute 1 (Supplementary Table 1).

Bottom frequency (Fig. 2e and Supplementary Fig. 1e) did not vary intratest in any stage (Fig. 2e); however, when comparing ages within each minute, we found that early larval zebrafish tend to entry the bottom zone of the tank less than late larval and juvenile animals throughout the test, except in the second and last minutes (Supplementary Table 1). This observation may be explained by the early larval fish showing a marked decrease in bottom time (Fig. 2f and Supplementary Fig. 1f) in the 5th minute of testing (minutes 3–5 *P*_Tukey_ = 0.00534; minutes 4–5 *P*_Tukey_ = 0.0152), which, in conjunction with the bottom frequency data, suggests that early larval fish entered and remained in the top zone during this time (Fig. 2f). Bottom time, when compared between stages within each minute, also corroborates the findings of the total test time, where adult fish had the highest bottom time, especially compared with late juveniles, which had the lowest bottom time (Supplementary Table 1).

Lastly, turn angle (Fig. 2g and Supplementary Fig. 1g) did not vary between minutes within the same developmental stage (Fig. 2g), but the increase in turn angle observed while analyzing total turn angle in early larval fish was sustained throughout the test in all minutes when compared with every other stage (Supplementary Fig. 1g; *P*_Tukey_ < 0.0001 for all comparisons).

### Social interaction test

**Fig. 3:**
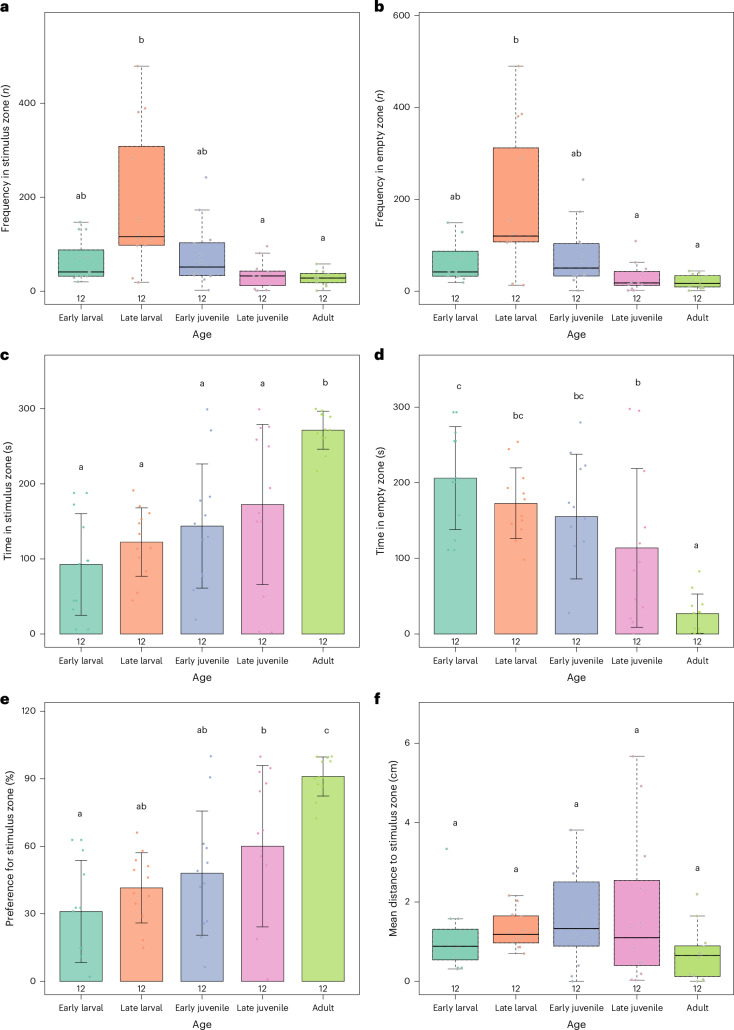
Social interaction parameters of zebrafish behavior across life stages. **a**, Frequency in the stimulus zone (*n*). **b**, Frequency in empty zone (*n*). **c**, Time in stimulus zone (s). **d**, Time in empty zone (s). **e**, Preference for the stimulus zone (%)). **f**, Mean distance to stimulus zone (cm)). Data for **a**, **b** and **f** were analyzed by Kruskal–Wallis followed by Dunn’s post hoc test, and are expressed as median ± IQR, whereas data for **c**, **d** and **e** were analyzed by one-way ANOVA followed by Tukey’s post hoc test and are expressed as mean ± s.d. Animals from both sexes were analyzed at distinct developmental stages: Early larval (7 dpf), mid-larval (14 dpf), late larval (21 dpf), early juvenile (~30 dpf), late juvenile (~45 dpf) and adult (>90 dpf). Anatomical characteristics pertaining to age and sex are detailed in Table 1. Sample sizes are shown in the figure. Different letters demonstrate statistically different groups. Detailed statistical values are provided in ‘Results’ and Supplementary Table 1.

Sociability is a key innate quality of zebrafish as a model. However, the timing of the onset of social behavior in this species remains a subject of debate in the literature. Social behavior in fish encompasses a series of phenotypes that span from social recognition of conspecifics to shoaling and aggressive phenotypes. Here, we investigated social recognition and preference (social interaction; Fig. 3). Data were analyzed either by one-way ANOVA with Tukey post hoc test or by Kruskal–Wallis test with Dunn post hoc test, depending on the results of normality testing. Frequency spent in the stimulus side (Fig. 3a; *H* = 18.1743; *P* = 0.0011) showed that late larval fish tended to have more entries in the stimulus zone than late juveniles (*P*_Dunn_ = 0.0058) or adults (*P*_Dunn_ = 0.0018). Frequency in the empty zone (Fig. 3b; *H* = 21.6526; *P* = 0.0002) also showed this pattern, with late larval fish showing a higher frequency in this zone compared to late juveniles (*P*_Dunn_ = 0.0005) and adults (*P*_Dunn_ = 0.0037).

Regarding the time in the stimulus zone (Fig. 3c; *F*_(4,55)_ = 11.0614, *P* < 0.0001), only adult fish show an increase in the time spent in this zone compared to all other stages (compared with early larval *P*_Tukey_ < 0.0001; late larval *P*_Tukey_ < 0.0001; early juvenile *P*_Tukey_ = 0.0005; late juvenile *P*_Tukey_ = 0.0125). However, the analysis of time spent in the empty zone (Fig. 3d; *F*_(4,55)_ = 11.2898, *P* < 0.0001) demonstrated that not only adult fish differed from all other stages (in comparison with: early larval *P*_Tukey_ < 0.0001; late larval *P*_Tukey_ < 0.0001; early juvenile *P*_Tukey_ = 0.0004; late juvenile *P*_Tukey_ = 0.0403), but the early larval stage also differed from the late juvenile stage (*P*_Tukey _= 0.0240). This pattern was also observed when preference for the stimulus zone (Fig. 3e; *F*_(4,55)_ = 11.0632, *P* < 0.0001) was evaluated: adult fish differed from the other stages (in comparison with: early larval *P*_Tukey_ < 0.0001; late larval *P*_Tukey_ < 0.0001; early juvenile *P*_Tukey_ = 0.0005; late juvenile *P*_Tukey_ = 0.0252), and late juveniles showed a higher preference for conspecifics than early larvae (*P*_Tukey_ = 0.0444).

We also analyzed the mean distance to stimulus zone (Fig. 3f) and found no differences between ages.

### Light/dark scototaxis test

**Fig. 4:**
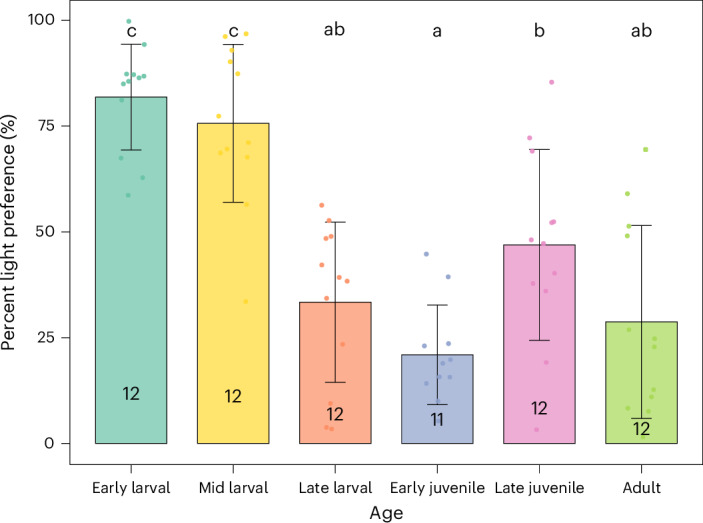
Scototaxis (light preference %) in zebrafish across life stages. Data were analyzed by one-way ANOVA followed by Tukey’s post hoc test. Animals from both sexes were analyzed at distinct developmental stages: early larval (7 dpf), mid-larval (14 dpf), late larval (21 dpf), early juvenile (~30 dpf), late juvenile (~45 dpf) and adult (>90 dpf). Anatomical characteristics pertaining to age and sex are detailed in Table 1. Data are expressed as mean ± s.d. Sample sizes are shown in the figure. Different letters demonstrate statistically different groups. Detailed statistical values are provided in ‘Results’ and Supplementary Table 1.

The light/dark test is commonly used as a measure of anxiety, stress or fear in zebrafish. Given that our data for the novel tank test indicate that the most used parameter of anxiety in adults (bottom time) cannot be translated to other life stages, we turned to the light/dark test as an alternative that could, potentially, be used across the different developmental stages. We adapted previous protocols that use a black/white background color (see Methods for details) for use across life stages. In adult zebrafish, a natural preference for the dark compartment has been consistently reported under standard light/dark test conditions. Thus, increased time spent in the light compartment may suggest altered anxiety-like behavior^36^. Early larval zebrafish, however, prefer lighter environments^11^, which changes the interpretation of results for this age. Because the exact stage of the reversal of scototaxis was not evaluated until now, we analyzed the preference for the light area for the early larval, late larval, early juvenile, late juvenile and adult stages using one-way ANOVA followed by Tukey post-hoc test (Fig. 4; *F*_(5,65)_ = 22.2508, *P* < 0.0001). In the first analysis, we found that the reversal of scototaxis had already occurred by the late larval stage (approximately 21 dpf). To observe the temporal window in which this shift occurs, a mid-larval stage was included only for this assay, with animals from two independent breeding sessions and two different breeding pairs tested at 14 dpf. The results demonstrate that early larvae show a high preference for the light side compared with the late larval (*P*_Tukey_ < 0.0001), early juvenile (*P*_Tukey_ = 0.0191), late juvenile (*P*_Tukey_ = 0.0003) and adult (*P*_Tukey_ < 0.0001) stages. Mid larvae had a similar pattern, differing from late larvae (*P*_Tukey_ < 0.0001), early juveniles (*P*_Tukey_ < 0.0001), late juveniles (*P*_Tukey_ = 0.0047) and adults (*P*_Tukey_ < 0.0001). Moreover, early juveniles had reduced preference for the light side compared with late juveniles (*P*_Tukey_ = 0.0191).

## Discussion

This work reports on the use of a new behavioral analysis platform to evaluate behavior of zebrafish across life stages. Using this platform, we have shown the first comparison of locomotor, social and scototaxis behaviors in larval, juvenile and adult zebrafish. Our platform, adapted from the novel tank test that is commonly used for adult zebrafish, enabled a direct and standardized comparison of behavioral phenotypes—including those observed during the juvenile stages—within a single testing framework across all life stages. While previous studies^37^ examined behavioral development using different setups in each stage, our approach applied a single, standardized testing environment across larval, juvenile and adult phases, allowing direct comparisons across ontogeny.

Juvenile behavior observed in our locomotion analysis seems to support the initial hypothesis proposed by Varga and collaborators^34^, suggesting that the morphological transition from larvae to juvenile fish is accompanied by a behavioral metamorphosis. Fish in the late larval and early juvenile stages showed an increase in the traveled distance and a decrease in immobility, compared with other life stages. Late larval zebrafish also showed an increase in the frequency in the top zone, while no changes in the time in this zone were observed. Moreover, both juvenile stages analyzed displayed a reduction in erratic movements, measured by the turn-angle parameter and immobility. It is important to note, however, that many of these changes occur in a pervasive manner, becoming more evident as the stage gaps under comparison become more distinct from one another, indicating that the behavioral metamorphosis takes place in a slow pace throughout zebrafish’s adolescence.

Our findings suggest that the preference for the bottom zone of the tank is an innate response to novel environments in zebrafish; consequently, all ages spent more time in the bottom than in the top zone during our tests. For adults, this is an established phenomena^11^, and it has recently been also demonstrated for 7-dpf-old larvae^38^. Evaluating larval behavior in rectangular tanks has only recently been tried, using an apparatus substantially different from ours (with a higher water column and smaller width). The persistence of the preference for the bottom zone in both our study and Fontana’s and Parker’s study^38^ using the larval diving response test supports the concept that bottom preference of larval zebrafish is innate. Furthermore, our results suggest that this preference may be a life-long trait of the species.

In adult zebrafish, the novel tank test is often analyzed per-minute intervals, and animals display a habituation to the novel environment in this stage, exploring it gradually more as the time of testing elapses^11^. In our analysis, while we did not observe the expected increase in top time at the last minutes of testing in adult zebrafish, we did observe a reduction in immobile time from the 3rd minute onward and an increase in top frequency from the 2nd minute onward, which we consider to be more subtle signs of habituation. For early larval fish, we observed an increase in time in the top section of the tank in the last minute of our 5-min session. This result is considerably different from the previous report for the matched stage, where 7-dpf larvae showed such responses only after 30 min of testing^38^. As we discussed previously, the tank shape difference is also relevant in this context.

Time spent in the bottom zone and increased exploration of the tank as test time elapses are key parameters for evaluating anxiety in the novel tank test in zebrafish^39^. To ascertain anxiety-like phenotypes in this test, control fish must show a preference for the bottom zone but gradually explore more the top zone during the testing session. On the other hand, anxious fish tend to stay in the bottom zone and have less transitions to the upper half of the tank^39^. While our results for early larval and adult fish corroborate the usefulness of our adaptation of the novel tank test for use across life stages for the evaluation of anxiety, our results for late larval and both juvenile stages do not meet these conditions. Our main hypothesis is that zebrafish, like mammals^20,21^, may be more prone to novelty seeking and risk-taking in the juvenile stage, which is corroborated by the reduced time spent in the bottom zone across all the testing intervals (minutes). This hypothesis must, however, be further investigated in dedicated tests, such as boldness and novel-object recognition^40,41^, in the future.

Our results for naive larvae and adult fish suggest a hypothesis in which behavior could be tested in early larvae and somewhat extrapolated to adulthood—but not for the juvenile period—as the differences between stages are negligible in some cases. However, determining whether the behavioral similarities observed between larvae and adult reflect the same underlying mechanisms, therefore leading to the same outcomes, requires further studies of neural markers and responses to known pharmacological interventions.

Given that our anxiety parameters in the juvenile novel tank test were not fully reliable, we also investigated how juvenile zebrafish behaved in the scototaxis test. This is the most widely used test to evaluate anxiety in zebrafish, and many methodological variations exist. Previous studies have used the test in both larval and adult zebrafish^42–44^, showing that larvae zebrafish preferred and remained more time in the white compartment of the apparatus, while adult fish preferred and remained more time in the dark compartment^43,45,46^. Thus, we aimed to investigate when this reversal of scototaxis occurred and if the preference was stable once it had been reversed. Surprisingly, when we analyzed fish preference for dark or light, we observed that the reversal for dark preference had already happened in the late larval stage, at approximately 21 dpf, and persisted to adulthood. In addition, we analyzed an intermediate stage of larval fish, at 14 dpf, which, similar to the early larval stage, spent more time in the light compartment than in the dark compartment. This analysis allowed us to conclude that reversal of scototaxis in zebrafish takes place between ~14 dpf and ~21 dpf, possibly being one of the first signs of the behavioral metamorphosis between larval and juvenile fish. Moreover, our results indicate that juveniles’ behavior in the scototaxis test is stable enough to be used for investigating anxiety in this life stage.

Lastly, to further understand zebrafish juvenile behavior, we also performed a social interaction test across all ages. The social domain is extremely relevant in mammal adolescence, with deficits linked to psychopathologies^19^. However, conflicting results remain regarding when social behavior develops in zebrafish. The social domain in zebrafish covers social recognition and interaction to conspecifics, the formation of social hierarchies and shoals, aggression and sexual behaviors, all traits that are highly conserved^11,47–49^. Nonsexual interaction comprises behaviors driven by a preference for conspecifics and, around 49 dpf, culminates in organized group swimming (shoaling)^48,50–52^. Regarding the age at which preference behaviors emerge, conflicting results exist in the literature. Factors influencing these results and that are potentially critical for the observed age differences include the number of fish tested and the stimulus fish used, size matching (or lack thereof) between test and stimulus fish, and the housing conditions of the animals before testing^48,51,53^. In addition, as observed for other behaviors in early developmental stages, body length rather than age in days seems to be a better predictor of neurodevelopment and, consequently, behavioral outcomes^48^. In our test, we observed a gradual increase in preference for the stimulus zone across life stages, with every stage showing a slight increase in comparison with the prior stage. A greater increase was seen in our late juvenile stage, supporting the concept that social capabilities are fully developed at this stage^50^. Our results seem to contradict previous reports that showed social preference in 3-week-old larvae^51^. However, Dreosti and colleagues^51^ reported that not all fish at this age displayed social preference, with some fish even displaying avoidance. In our analogous stage, the late larval stage, we observed an increase in the frequency in the empty zone of the tank, similar to what was previously observed, but at a higher rate in our work. As already stated, feeding, temperature and density are key to behavioral phenotypes at such young ages of zebrafish development. Given that they are not explicitly reported in the literature for comparison, we assume that some or all of these parameters could be responsible for the observed differences between results. Nonetheless, it seems that social recognition starts at approximately 3 weeks of life depending on rearing conditions. Then, after a brief period of social avoidance, social preference subtly increases until adulthood in zebrafish.

Overall, our results demonstrate that the novel tank test can be adapted for all ages of zebrafish development but should be approached with caution when assessing anxiety in the juvenile stages. This limitation could be mitigated with further studies that test the construct validity of this adaptation in the juvenile stage using known anxiogenic and anxiolytic drugs. We also demonstrated the significance of behavioral metamorphosis in juvenile zebrafish, which encompasses changes in locomotor parameters, scototaxis and social interaction.

## Conclusion

In this study, we adapted the adult zebrafish novel tank test, social interaction test and scototaxis test to develop a behavioral platform that allows comparisons between different life stages of the zebrafish model. In doing so, we also investigated the natural behavior of naive fish in its juvenile stages, which is so far poorly characterized in the literature. The use of a standardized platform, such as the one presented, and the initial characterization of juvenile behaviors will enable further efforts to conduct longitudinal studies of behavior in the zebrafish model.

## Methods

### Animals

Animals used in this study were obtained from breeding of wild-type AB strain adult zebrafish at the Center for Experimental Biological Models of our University (CeMBE – PUCRS). After breeding, 24-h-old eggs were bleached^54^ to avoid contaminants and allow entrance in the recirculating water system (Zebtec, Tecniplast) at 7 dpf. Water parameters were maintained at optimal levels for the species (temperature 26 °C ± 2 °C, pH 7.0–7.5, conductivity 300–700 μS, ammonia <0.02 mg/L, hardness 80–300 mg/L, nitrite <1 mg/L, nitrate <50 mg/L and chloride 0 mg/L)^55^. Density, water flux and feeding type varied across developmental stages (Supplementary Information). Animals were maintained in the recirculating system until behavioral assessment days, according to the developmental stages observed in this study: early larval stage, late larval stage, early juvenile stage, late juvenile stage and adult stage. Adult zebrafish of mixed sex were used in the experiments, with an aproximate ratio of 50% males and 50% females. Daily, during feeding, animals were checked for sickness behaviors and cryoeuthanized by immersion in freezing water if necessary.

These stages were selected based on an approximation of most used ages described in the literature (7, 21, 30, 45 and 120 dpf). However, for zebrafish, previous studies have shown that factors such as temperature, density, stress and others can influence developmental rates^14^, with animals that have the same age but different morphologies showing distinct behavioral endpoints^51^. For this reason, rather than assuming developmental stages by dpf, anatomical parameters were used to assign animals to groups. In short, animal size, swim bladder inflation, pigmentation, scales, fins and sexual dimorphism were used as endpoints^28,30^. The complete list of criteria can be found in Table 1.

Initially, groups of 30 animals of different ages and separate tanks were visually assessed for the characteristics. Once the approximate ages (7 dpf, 21 dpf, 28–32 dpf, 42–48 dpf and more than 90 dpf) were established for the set of characteristics under our lab and rearing conditions, animals were quickly checked on each experimental day while being captured from home tank. If any animal did not display the intended morphological traits or had other visual morphological defects, they were excluded and were not tested in any of our behavioral analysis.

All protocols were approved by the Institutional Animal Care Committee from Pontifícia Universidade Católica do Rio Grande do Sul (CEUA-PUCRS, permit number 10617), and the study followed the guidelines of the National Animal Experimentation Control Council (CONCEA).

### Experimental design

On each testing day, animals were transferred to the testing room. All tests were conducted between 10:00 and 15:00. Animals were not fed the feeding routine directly before testing (7:00 feeding for fish tested in mornings; 12:00 feeding for fish tested in the afternoons). Experiments were conducted in, at least, duplicates of testing days and breeding groups. For each experiment day, animals of different home tanks were assessed. A total of 12 fish per group was used. Sample size was based on previous studies^56^.

All experiments were conducted in rectangular tanks, with sizes matched with fish size (Supplementary. Table 2), as it has been reported that tank size relative to fish size may influence behavior^50,57,58^.

### Novel tank and social interaction tests

The novel tank and social interaction tests were conducted sequentially, using the same animals^59^. For these tests, the setup consisted of a central tank (experimental fish tank) with an empty tank on one side and a tank containing six stimulus animals matched in age and size on the other side. A camera was positioned in front of the tank to record animals’ behavior. In the first phase of the test, when exploratory behavior was assessed, both sides of the central tank in contact with the stimulus and empty tanks were blocked with white partitions. The back wall of the tank was also covered with a white partition to facilitate analysis. The animals swam freely for 6 min. The video was then paused, and the partitions were removed. Then, a new video was started to evaluate social interaction with a duration of 5 min.

### Light/dark scototaxis test

There are various protocols and distinct apparatuses for the light/dark test. In this study, we used a protocol from Serra, Medalha and Mattioli^44^, and Steenbergen, Richardson and Champagne^27^, with some modifications. This protocol was chosen because it does not require the manual opening of partitions in the tank by the experimenter, thus reducing potential bias; it is also more suitable for small tanks for larvae. For this assessment, the external walls and base of rectangular tanks were wrapped with ethylene-vinyl acetate (EVA) foam, with half of the tank covered in white EVA and the other half in black EVA. The animals were placed individually at the center of the apparatus, on the dividing line between light and dark. A camera was positioned above the apparatus and recorded the movement of the animals for 6 min.

### Data collection and analysis

All videos obtained during the test were evaluated using Ethovision XT v.14 software (Noldus). Analyses were conducted using sample rates of 30 samples per second with detection settings set to ‘dynamic subtraction’, adjusting subject color relative to background and subject size accordingly. All tests where the error values ‘missing samples’ or ‘subject not found’ surpassed 20% were excluded from analysis.

For the novel tank test, the arena was divided in two equal-sized zones, a bottom zone and a top zone. Parameters retrieved from the Ethovision software were: total distance traveled (cm), immobile time (s), frequency (*n*) and time (s) in the top zone, frequency (*n*) and time (s) in the bottom zone and absolute turn angle (°). For immobile time, the start/stop velocity setup for larval and early juvenile stages was 0.06 cm/s and 0.01 cm/s; and 0.6 cm/s and 0.59 cm/s for late juvenile and adult stages. Data were retrieved and analyzed as both total test time and per-minute intervals^60,61^.

For the social interaction test and light/dark test, parameters were retrieved as the total of the last 5 min of testing. Social interaction arenas were subdivided in two zones: zone near the stimulus fish and zone far from the stimulus fish (near the empty tank). The parameters recorded were: frequency (*n*) and time (s) near stimulus, frequency (n) and time (s) far from stimulus, and mean distance (cm) from stimulus zone. The light/dark test was analyzed by retrieving the time (s) in the white side of the arena.

Distance-related parameters were weighted by length of arena size (weighted distance = software-retrieved distance (cm)/arena length (cm)) to allow comparisons between ages. Preference for stimulus zone (%) was calculated by the following formula: (time in stimulus zone/(time in stimulus zone + time in empty zone)) × 100. Percent light preference (%) was calculated by the following formula: (time in light zone/total time of test) × 100.

### Statistical analysis

After data retrieval, datasets were imported to R 4.4.1 and treated for removal of outliers using the interquartile range (IQR) method. Data normality was analyzed by Shapiro–Wilk test using the package ‘stats’^62^. Depending on the results, data across the entire test duration were analyzed by one-way ANOVA with Tukey post hoc test, with *P* values adjusted using the Šidák method (packages ‘stats’^62^ and ‘emmeans’^63^) or Kruskal–Wallis test with Dunn post hoc with *P* value adjustment using the Bonferroni method (packages ‘stats’^62^ and ‘FSA’^64^). Analysis of the novel tank test by minute intervals were conducted by two-way RM-ANOVA with ‘age’ as the between-subjects factor and ‘minute’ as the within-subjects (repeated measures) factor (package ‘afex’^65^). After performing a repeated-measures ANOVA, Tukey post hoc tests with P-value adjustment using the Šidák method (package ‘emmeans’^63^) were conducted, both to compare ages within each minute and minutes within each age. Results were considered significant when *P* ≤ 0.05 for all tests. Results were expressed as mean ± standard deviation (s.d.) or median and IQR. R packages ‘ggplot2’, ‘multcompView’ and ‘RcolorBrewer’ were used to produce graphs^66–68^.

### Reporting summary

Further information on research design is available in the Nature Portfolio Reporting Summary linked to this article.

## Online content

Any methods, additional references, Nature Portfolio reporting summaries, source data, extended data, supplementary information, acknowledgements, peer review information; details of author contributions and competing interests; and statements of data and code availability are available at 10.1038/s41684-026-01712-x.

## Supplementary information

Supplementary Information Supplmentary Methods, Fig. 1, and Tables 1 and 2.

Reporting Summary

## Data Availability

The data for all experiments are available at https://osf.io/4an63/.
