# Supplementary material for: Ontogeny of zebrafish behaviors: comparative evaluation of locomotor, social and anxiety parameters in larval, juvenile and adult stages
Source: Lab Anim (NY). 2026 Apr 7;55(5):172–80. doi: 10.1038/s41684-026-01712-x (PMC13143822; doi:10.1038/s41684-026-01712-x)
Supplement: Supplementary file 1 — Supplmentary Methods, Fig. 1, and Tables 1 and 2. [file 41684_2026_1712_MOESM1_ESM.pdf]

lab animal

Supplementary information

https://doi.org/10.1038/s41684-026-01712-x

Ontogeny of zebrafish behaviors: comparative evaluation of locomotor, social and anxiety parameters in larval, juvenile and adult stages

In the format provided by the authors and unedited

Supplementary Material

Ontogeny of Zebrafish Behaviors: Comparative evaluation of Locomotor, Social and Anxiety Parameters in Larval, Juvenile and Adult Stages

B. D. Petersen [a,b], G. Rodrigues [b], K. Liriel [b], L. Ferreira [b], C.D. Bonan [a,b]

[a] *Programa de Pós-Graduação em Medicina e Ciências da Saúde, Escola de Medicina, Pontifícia Universidade Católica do Rio Grande do Sul, Porto Alegre, RS, Brazil*

[b] *Laboratório de Neuroquímica e Psicofarmacologia, Escola de Ciências da Saúde e da Vida, Pontifícia Universidade Católica do Rio Grande do Sul, Porto Alegre, RS, Brazil*

Corresponding author: Carla Denise Bonan. Email: cbonan@pucrs.br

1. Supplementary Methods

1.1. Rearing Conditions across Life Stages

To ensure the growth of the animals, the density was gradually adjusted. At 7 dpf, the larvae were transferred to tanks in a recirculating system, with 30 larvae in 500 mL of water, without water flow. From 10 to 14 dpf, the water level was gradually adjusted to a final volume of 3.5 L. At 30 dpf, the animal density was adjusted to a final density of 5 animals per liter of water, and the water flow was set to a high (constant) level.

The feeding of the larvae began at 5 dpf. During the transfer of animals to the recirculating system, 50 mL of paramecium infusion (*Paramecium caudatum*, colony maintained with powdered milk, final density ~100 paramecia per mL) was added. In addition to this initial amount, the animals received 2.5 mL of paramecium infusion daily until 7 dpf. From then on, feeding with paramecia was maintained, accompanied by commercial feed (TetraMin Tropical Flake Fish®) ground in a mortar three times a day. From 10 to 14 dpf, the animals received supplementation with brine shrimp (Artemia salina) during the last feeding of the day. From 14 to 30 dpf, the animals were fed ground commercial feed three times a day, paramecia during the first feeding of the day, and supplementation with brine shrimp during the last feeding of the day. From 30 dpf onwards, feeding consisted of flaked commercial feed three times a day and supplementation with brine shrimp during the last feeding of the day.

2. Supplementary Results

2.1. Supplementary Figure 1

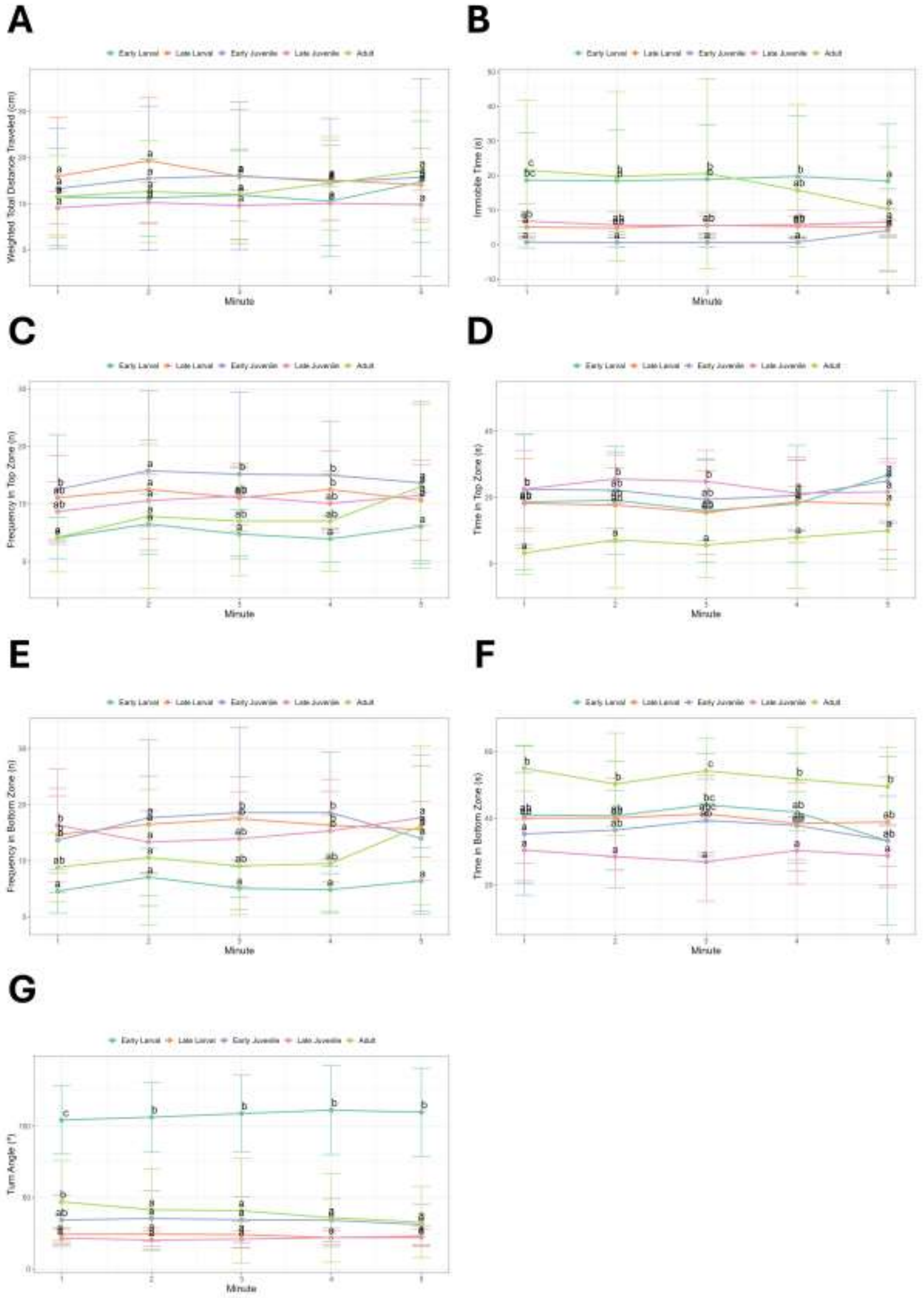
A
B
C
D
E
F
G
Early Larval
Late Larval
Early Juvenile
Late Juvenile
Adult
Weighted Total Distance Traveled (cm)
Immobile Time (s)
Frequency in Top Zone (n)
Time in Top Zone (s)
Frequency in Bottom Zone (n)
Time in Bottom Zone (s)
Turn Angle (°)
Minute

Supplementary Figure 1. Per-Minute intervals of zebrafish behavior on the Novel Tank Test across life-stages. (A) Weighted Distance Travelled (cm), (B) Immobile Time (s),(C) Frequency in the Top Tank Zone (n), (D) Time in the Top Tank Zone (s), (E) Frequency in the Bottom Tank Zone (n), (F) Time in the Bottom Tank Zone (s) and (G) Turn-angle (º). Data were analysed by RM-ANOVA followed by Tukey's post-hoc and are expressed in Mean ± SD. Different letters show significant differences between ages within each minute.

2.2. Supplementary Table 1

Supplementary Table 1 - Statistics

	Test	F	p-value	Post-hoc	Comparison		*P*-value
Novel Tank Test Turn Angle	RM-ANOVA	(4,54) = 45,555	1,08155E -16	Tukey	7dpf	1' - 2'	0,912234
					7dpf	1' - 3'	0,831924
					7dpf	1' - 4'	0,561012
					7dpf	1' - 5'	0,689025
					7dpf	2' - 3'	0,949938
					7dpf	2' - 4'	0,569107
					7dpf	2' - 5'	0,892986
					7dpf	3' - 4'	0,956228
					7dpf	3' - 5'	0,999109
					7dpf	4' - 5'	0,953796
					21dpf	1' - 2'	0,999999
					21dpf	1' - 3'	1
					21dpf	1' - 4'	0,994392
Novel Tank Test Turn Angle	RM-ANOVA	(4,54) = 45,555	1,08155E -16	Tukey	21dpf	1' - 5'	0,999365
					21dpf	2' - 3'	0,99997
					21dpf	2' - 4'	0,958981
					21dpf	2' - 5'	0,996772
					21dpf	3' - 4'	0,988475
					21dpf	3' - 5'	0,999558
					21dpf	4' - 5'	0,992977
					30dpf	1' - 2'	0,998876
					30dpf	1' - 3'	1
					30dpf	1' - 4'	1
					30dpf	1' - 5'	0,971213
					30dpf	2' - 3'	0,999008

					30dpf	2' - 4'	0,996871
					30dpf	2' - 5'	0,808335
					30dpf	3' - 4'	0,999981
					30dpf	3' - 5'	0,953086
					30dpf	4' - 5'	0,767491
					45dpf	1' - 2'	0,997442
					45dpf	1' - 3'	0,999941
					45dpf	1' - 4'	0,999992
					45dpf	1' - 5'	0,999985
					45dpf	2' - 3'	0,999742
					45dpf	2' - 4'	0,989334
					45dpf	2' - 5'	0,991767
					45dpf	3' - 4'	0,999029
					45dpf	3' - 5'	0,999294
					45dpf	4' - 5'	1
					120dpf	1' - 2'	0,586978
					120dpf	1' - 3'	0,763194
					120dpf	1' - 4'	0,286723
					120dpf	1' - 5'	0,057265
					120dpf	2' - 3'	0,999594
					120dpf	2' - 4'	0,50536
					120dpf	2' - 5'	0,174205
					120dpf	3' - 4'	0,772581
					120dpf	3' - 5'	0,452915
					120dpf	4' - 5'	0,757793
Novel Tank Test – Weighted Travelled Distance	RM-ANOVA	(4,54) = 0,815914	0,520630 11	Tukey	7dpf	1' - 2'	0,993586
					7dpf	1' - 3'	0,999997
					7dpf	1' - 4'	1
					7dpf	1' - 5'	0,932774
					7dpf	2' - 3'	0,997949
					7dpf	2' - 4'	0,994977
					7dpf	2' - 5'	0,985823
					7dpf	3' - 4'	0,999996
Novel Tank Test – Weighted Travelled Distance	RM-ANOVA	(4,54) = 0,815914	0,520630 11	Tukey	7dpf	3' - 5'	0,958006
					7dpf	4' - 5'	0,914188
					21dpf	1' - 2'	0,280841
					21dpf	1' - 3'	1
					21dpf	1' - 4'	0,988578
					21dpf	1' - 5'	0,964095
					21dpf	2' - 3'	0,41248
					21dpf	2' - 4'	0,198648
					21dpf	2' - 5'	0,402702
					21dpf	3' - 4'	0,996339
					21dpf	3' - 5'	0,974447
					21dpf	4' - 5'	0,994185
					30dpf	1' - 2'	0,690221

					30dpf	1' - 3'	0,533127
					30dpf	1' - 4'	0,890118
					30dpf	1' - 5'	0,910426
					30dpf	2' - 3'	0,998289
					30dpf	2' - 4'	0,995155
					30dpf	2' - 5'	0,999994
					30dpf	3' - 4'	0,97269
					30dpf	3' - 5'	0,999955
					30dpf	4' - 5'	0,9965
					45dpf	1' - 2'	0,956968
					45dpf	1' - 3'	0,998631
					45dpf	1' - 4'	0,970919
					45dpf	1' - 5'	0,999086
					45dpf	2' - 3'	0,99674
					45dpf	2' - 4'	0,999984
					45dpf	2' - 5'	0,999858
					45dpf	3' - 4'	0,999296
					45dpf	3' - 5'	0,999995
					45dpf	4' - 5'	0,999965
					120dpf	1' - 2'	0,966934
					120dpf	1' - 3'	0,99904
					120dpf	1' - 4'	0,398353
					120dpf	1' - 5'	0,290997
					120dpf	2' - 3'	0,997435
					120dpf	2' - 4'	0,883344
					120dpf	2' - 5'	0,561039
					120dpf	3' - 4'	0,792229
					120dpf	3' - 5'	0,465075
					120dpf	4' - 5'	0,846968
Novel Tank Test – Bottom Frequency	RM-ANOVA	(4,54) = 4,386489463	0,003827134	Tukey	7dpf	1' - 2'	0,909652
					7dpf	1' - 3'	1
					7dpf	1' - 4'	0,999911
					7dpf	1' - 5'	0,992209
Novel Tank Test – Bottom Frequency	RM-ANOVA	(4,54) = 4,386489463	0,003827134	Tukey	7dpf	2' - 3'	0,850209
					7dpf	2' - 4'	0,825198
					7dpf	2' - 5'	0,99816
					7dpf	3' - 4'	0,999905
					7dpf	3' - 5'	0,993075
					7dpf	4' - 5'	0,980906
					21dpf	1' - 2'	0,94193
					21dpf	1' - 3'	0,742533
					21dpf	1' - 4'	0,898794
					21dpf	1' - 5'	0,997401
					21dpf	2' - 3'	0,988615
					21dpf	2' - 4'	0,999992
					21dpf	2' - 5'	0,997748

					21dpf	3' - 4'	0,968777
					21dpf	3' - 5'	0,962726
					21dpf	4' - 5'	0,99859
					30dpf	1' - 2'	0,473378
					30dpf	1' - 3'	0,231269
					30dpf	1' - 4'	0,078946
					30dpf	1' - 5'	0,999952
					30dpf	2' - 3'	0,991813
					30dpf	2' - 4'	0,993659
					30dpf	2' - 5'	0,711989
					30dpf	3' - 4'	1
					30dpf	3' - 5'	0,507883
					30dpf	4' - 5'	0,431904
					45dpf	1' - 2'	0,729978
					45dpf	1' - 3'	0,837227
					45dpf	1' - 4'	0,983297
					45dpf	1' - 5'	0,989037
					45dpf	2' - 3'	0,998578
					45dpf	2' - 4'	0,894374
					45dpf	2' - 5'	0,568365
					45dpf	3' - 4'	0,938028
					45dpf	3' - 5'	0,689965
					45dpf	4' - 5'	0,904991
					120dpf	1' - 2'	0,950613
					120dpf	1' - 3'	0,999969
					120dpf	1' - 4'	0,996433
					120dpf	1' - 5'	0,073754
					120dpf	2' - 3'	0,949931
					120dpf	2' - 4'	0,988037
					120dpf	2' - 5'	0,299924
					120dpf	3' - 4'	0,999397
					120dpf	3' - 5'	0,107613
					120dpf	4' - 5'	0,098983
Novel Tank Test – Bottom Time	RM-ANOVA	(4,54) = 6,296932302	0,000310885	Tukey	7dpf	1' - 2'	0,990753
					7dpf	1' - 3'	0,84049
					7dpf	1' - 4'	0,979555
					7dpf	1' - 5'	0,415695
					7dpf	2' - 3'	0,92594
					7dpf	2' - 4'	0,99973
					7dpf	2' - 5'	0,133542
					7dpf	3' - 4'	0,970775
					7dpf	3' - 5'	0,005342
					7dpf	4' - 5'	0,015228
					21dpf	1' - 2'	1
					21dpf	1' - 3'	0,996653
					21dpf	1' - 4'	0,994369

					21dpf	1' - 5'	0,999123
					21dpf	2' - 3'	0,987661
					21dpf	2' - 4'	0,987832
					21dpf	2' - 5'	0,998352
					21dpf	3' - 4'	0,671438
					21dpf	3' - 5'	0,944766
					21dpf	4' - 5'	0,999855
					30dpf	1' - 2'	0,997865
					30dpf	1' - 3'	0,783723
					30dpf	1' - 4'	0,972046
					30dpf	1' - 5'	0,991859
					30dpf	2' - 3'	0,756058
					30dpf	2' - 4'	0,994446
					30dpf	2' - 5'	0,93429
					30dpf	3' - 4'	0,952995
					30dpf	3' - 5'	0,331278
					30dpf	4' - 5'	0,603589
					45dpf	1' - 2'	0,982788
					45dpf	1' - 3'	0,860836
					45dpf	1' - 4'	1
					45dpf	1' - 5'	0,995898
					45dpf	2' - 3'	0,970714
					45dpf	2' - 4'	0,982865
					45dpf	2' - 5'	0,999995
					45dpf	3' - 4'	0,532076
					45dpf	3' - 5'	0,979941
					45dpf	4' - 5'	0,987395
					120dpf	1' - 2'	0,699068
					120dpf	1' - 3'	0,999461
					120dpf	1' - 4'	0,929199
					120dpf	1' - 5'	0,756412
					120dpf	2' - 3'	0,494046
					120dpf	2' - 4'	0,993043
					120dpf	2' - 5'	0,999744
Novel Tank Test – Bottom Time	RM-ANOVA	(4,54) = 6,296932302	0,000310885	Tukey			
					120dpf	3' - 4'	0,778338
					120dpf	3' - 5'	0,591999
					120dpf	4' - 5'	0,956583
Novel Tank Test – Top Frequency	RM-ANOVA	(4,54) = 2,814510573	0,034054122	‘	7dpf	1' - 2'	0,808117
					7dpf	1' - 3'	0,999867
					7dpf	1' - 4'	0,99227
					7dpf	1' - 5'	0,986643
					7dpf	2' - 3'	0,852654
					7dpf	2' - 4'	0,632557
					7dpf	2' - 5'	0,997996
					7dpf	3' - 4'	0,982358

					7dpf	3' - 5'	0,994809
					7dpf	4' - 5'	0,921075
					21dpf	1' - 2'	0,952599
					21dpf	1' - 3'	1
					21dpf	1' - 4'	0,847866
					21dpf	1' - 5'	0,999845
					21dpf	2' - 3'	0,94546
					21dpf	2' - 4'	1
					21dpf	2' - 5'	0,966351
					21dpf	3' - 4'	0,901727
					21dpf	3' - 5'	0,99986
					21dpf	4' - 5'	0,931881
					30dpf	1' - 2'	0,481492
					30dpf	1' - 3'	0,515706
					30dpf	1' - 4'	0,390298
					30dpf	1' - 5'	0,991267
					30dpf	2' - 3'	0,998042
					30dpf	2' - 4'	0,995622
					30dpf	2' - 5'	0,947074
					30dpf	3' - 4'	0,999973
					30dpf	3' - 5'	0,979622
					30dpf	4' - 5'	0,978053
					45dpf	1' - 2'	0,869599
					45dpf	1' - 3'	0,515706
					45dpf	1' - 4'	0,847866
					45dpf	1' - 5'	0,809104
					45dpf	2' - 3'	0,994809
					45dpf	2' - 4'	0,999101
					45dpf	2' - 5'	0,997548
					45dpf	3' - 4'	0,935414
					45dpf	3' - 5'	0,999996
					45dpf	4' - 5'	0,972603
					120dpf	1' - 2'	0,358701
					120dpf	1' - 3'	0,454658
					120dpf	1' - 4'	0,266917
Novel Tank Test – Top Frequency	RM-ANOVA	(4,54) = 2,814510573	0,034054122	Tukey	120dpf	1' - 5'	0,010441
					120dpf	2' - 3'	0,992228
					120dpf	2' - 4'	0,993435
					120dpf	2' - 5'	0,370044
					120dpf	3' - 4'	1
					120dpf	3' - 5'	0,17475
					120dpf	4' - 5'	0,086206
Novel Tank Test – Top Time	RM-ANOVA	(4,54) = 4,006992148	0,006432939	Tukey	7dpf	1' - 2'	0,994917
					7dpf	1' - 3'	0,87249
					7dpf	1' - 4'	0,988403
					7dpf	1' - 5'	0,357224

					7dpf	2' - 3'	0,917717
					7dpf	2' - 4'	0,999834
					7dpf	2' - 5'	0,11998
					7dpf	3' - 4'	0,978651
					7dpf	3' - 5'	0,005578
					7dpf	4' - 5'	0,01818
					21dpf	1' - 2'	0,999896
					21dpf	1' - 3'	0,923841
					21dpf	1' - 4'	0,999926
					21dpf	1' - 5'	0,999998
					21dpf	2' - 3'	0,851373
					21dpf	2' - 4'	0,998134
					21dpf	2' - 5'	0,999996
					21dpf	3' - 4'	0,616638
					21dpf	3' - 5'	0,934982
					21dpf	4' - 5'	0,999212
					30dpf	1' - 2'	0,999994
					30dpf	1' - 3'	0,891167
					30dpf	1' - 4'	0,98956
					30dpf	1' - 5'	0,980075
					30dpf	2' - 3'	0,716751
					30dpf	2' - 4'	0,989562
					30dpf	2' - 5'	0,955435
					30dpf	3' - 4'	0,984234
					30dpf	3' - 5'	0,411627
					30dpf	4' - 5'	0,647518
					45dpf	1' - 2'	0,901942
					45dpf	1' - 3'	0,958479
					45dpf	1' - 4'	0,997809
					45dpf	1' - 5'	0,999756
					45dpf	2' - 3'	0,997373
					45dpf	2' - 4'	0,715898
					45dpf	2' - 5'	0,867364
					45dpf	3' - 4'	0,542666
					45dpf	3' - 5'	0,864407
					45dpf	4' - 5'	0,999932
Novel Tank Test – Top Time	RM-ANOVA	(4,54) = 4,006992148	0,006432939	Tukey	120dpf	1' - 2'	0,776762
					120dpf	1' - 3'	0,95375
					120dpf	1' - 4'	0,754992
					120dpf	1' - 5'	0,576993
					120dpf	2' - 3'	0,951807
					120dpf	2' - 4'	0,999584
					120dpf	2' - 5'	0,958271
					120dpf	3' - 4'	0,854487
					120dpf	3' - 5'	0,657333
					120dpf	4' - 5'	0,968295

Novel Tank Test – Immobile Time	RM-ANOVA	(4,54) = 5,692826281	0,000674395	Tukey	7dpf	1' - 2'	0,996543
					7dpf	1' - 3'	0,986519
					7dpf	1' - 4'	0,922881
					7dpf	1' - 5'	0,997095
					7dpf	2' - 3'	0,986909
					7dpf	2' - 4'	0,865027
					7dpf	2' - 5'	1
					7dpf	3' - 4'	0,996985
					7dpf	3' - 5'	0,999709
					7dpf	4' - 5'	0,979931
					21dpf	1' - 2'	0,999974
					21dpf	1' - 3'	0,999773
					21dpf	1' - 4'	0,999993
					21dpf	1' - 5'	1
					21dpf	2' - 3'	0,964904
					21dpf	2' - 4'	0,997136
					21dpf	2' - 5'	0,999989
					21dpf	3' - 4'	0,999895
					21dpf	3' - 5'	0,99976
					21dpf	4' - 5'	0,999974
					30dpf	1' - 2'	1
					30dpf	1' - 3'	1
					30dpf	1' - 4'	1
					30dpf	1' - 5'	0,745247
					30dpf	2' - 3'	1
					30dpf	2' - 4'	1
					30dpf	2' - 5'	0,73012
					30dpf	3' - 4'	1
					30dpf	3' - 5'	0,831602
					30dpf	4' - 5'	0,643523
					45dpf	1' - 2'	0,992591
					45dpf	1' - 3'	0,99395
					45dpf	1' - 4'	0,996277
					45dpf	1' - 5'	0,999952
					45dpf	2' - 3'	0,999977
					45dpf	2' - 4'	0,999992
					45dpf	2' - 5'	0,998452
Novel Tank Test – Immobile Time	RM-ANOVA	(4,54) = 5,692826281	0,000674395	Tukey	45dpf	3' - 4'	0,999973
					45dpf	3' - 5'	0,998539
					45dpf	4' - 5'	0,998628
					120dpf	1' - 2'	0,952382
					120dpf	1' - 3'	0,997605
					120dpf	1' - 4'	0,255953
					120dpf	1' - 5'	0,001558
					120dpf	2' - 3'	0,952708
					120dpf	2' - 4'	0,059932

					120dpf	2' - 5'	0,012434
					120dpf	3' - 4'	0,225017
					120dpf	3' - 5'	0,022795
					120dpf	4' - 5'	0,205683
Novel Tank Test – Turn Angle	RM-ANOVA	(4,54) = 45,55521852	1,08155E -16	Tukey	1	7dpf - 21dpf	1,05E-12
					1	7dpf - 30dpf	5,75E-11
					1	7dpf - 45dpf	6,51E-13
					1	7dpf - 120dpf	2,21E-08
					1	21dpf - 30dpf	0,688861
					1	21dpf - 45dpf	0,996947
					1	21dpf - 120dpf	0,036401
					1	30dpf - 45dpf	0,473983
					1	30dpf - 120dpf	0,4772
					1	45dpf - 120dpf	0,014563
					2	7dpf - 21dpf	7,89E-13
					2	7dpf - 30dpf	4,06E-11
					2	7dpf - 45dpf	5,24E-13
					2	7dpf - 120dpf	6,51E-10
					2	21dpf - 30dpf	0,64014
					2	21dpf - 45dpf	0,98591
					2	21dpf - 120dpf	0,21107
					2	30dpf - 45dpf	0,33212
					2	30dpf - 120dpf	0,936871
					2	45dpf - 120dpf	0,071988
					3	7dpf - 21dpf	1,32E-11
					3	7dpf - 30dpf	7,92E-10
					3	7dpf - 45dpf	4,25E-12
					3	7dpf - 120dpf	9,82E-09
					3	21dpf - 30dpf	0,77824
					3	21dpf - 45dpf	0,996887
					3	21dpf - 120dpf	0,359969
					3	30dpf - 45dpf	0,569446
					3	30dpf - 120dpf	0,957435
					3	45dpf - 120dpf	0,199895
					4	7dpf - 21dpf	7,29E-13
					4	7dpf - 30dpf	3,58E-11
					4	7dpf - 45dpf	7,34E-13
					4	7dpf - 120dpf	7,23E-11
Novel Tank Test – Turn Angle	RM-ANOVA	(4,54) = 45,55521852	1,08155E -16	Tukey	4	21dpf - 30dpf	0,624295
					4	21dpf - 45dpf	1
					4	21dpf - 120dpf	0,499233
					4	30dpf - 45dpf	0,627712
					4	30dpf - 120dpf	0,999646
					4	45dpf - 120dpf	0,502613
					5	7dpf - 21dpf	5,12E-13
					5	7dpf - 30dpf	1,84E-12

					5	7dpf - 45dpf	4,93E-13
					5	7dpf - 120dpf	3,31E-12
					5	21dpf - 30dpf	0,845564
					5	21dpf - 45dpf	0,999939
					5	21dpf - 120dpf	0,735011
					5	30dpf - 45dpf	0,781248
					5	30dpf - 120dpf	0,999558
					5	45dpf - 120dpf	0,658046
Novel Tank Test – Weighted Travelled Distance	RM-ANOVA	(4,54) = 0,815913909	0,520630 11	Tukey	1	7dpf - 21dpf	0,707972
					1	7dpf - 30dpf	0,963794
					1	7dpf - 45dpf	0,997949
					1	7dpf - 120dpf	0,999003
					1	21dpf - 30dpf	0,970098
					1	21dpf - 45dpf	0,492073
					1	21dpf - 120dpf	0,83582
					1	30dpf - 45dpf	0,857715
					1	30dpf - 120dpf	0,993735
					1	45dpf - 120dpf	0,977557
					2	7dpf - 21dpf	0,433739
					2	7dpf - 30dpf	0,891642
					2	7dpf - 45dpf	0,999719
					2	7dpf - 120dpf	0,997967
					2	21dpf - 30dpf	0,92259
					2	21dpf - 45dpf	0,30705
					2	21dpf - 120dpf	0,606493
					2	30dpf - 45dpf	0,794242
					2	30dpf - 120dpf	0,972033
					2	45dpf - 120dpf	0,986366
					3	7dpf - 21dpf	0,793415
					3	7dpf - 30dpf	0,774028
					3	7dpf - 45dpf	0,999657
					3	7dpf - 120dpf	0,998047
					3	21dpf - 30dpf	1
					3	21dpf - 45dpf	0,661803
					3	21dpf - 120dpf	0,918032
					3	30dpf - 45dpf	0,63889
					3	30dpf - 120dpf	0,905478
					3	45dpf - 120dpf	0,985695
					4	7dpf - 21dpf	0,805532
Novel Tank Test – Weighted Travelled Distance	RM-ANOVA	(4,54) = 0,815913909	0,520630 11	Tukey	4	7dpf - 30dpf	0,847723
					4	7dpf - 45dpf	0,999996
					4	7dpf - 120dpf	0,881991
					4	21dpf - 30dpf	0,999986
					4	21dpf - 45dpf	0,75561
					4	21dpf - 120dpf	0,99981
					4	30dpf - 45dpf	0,803517

					4	30dpf - 120dpf	0,99999
					4	45dpf - 120dpf	0,843508
					5	7dpf - 21dpf	0,999268
					5	7dpf - 30dpf	0,981836
					5	7dpf - 45dpf	0,981654
					5	7dpf - 120dpf	0,925558
					5	21dpf - 30dpf	0,997766
					5	21dpf - 45dpf	0,928206
					5	21dpf - 120dpf	0,976332
					5	30dpf - 45dpf	0,795092
					5	30dpf - 120dpf	0,998766
					5	45dpf - 120dpf	0,635293
					1	7dpf - 21dpf	0,026631
					1	7dpf - 30dpf	0,058915
					1	7dpf - 45dpf	0,006104
					1	7dpf - 120dpf	0,731531
					1	21dpf - 30dpf	0,997613
					1	21dpf - 45dpf	0,983173
					1	21dpf - 120dpf	0,342416
					1	30dpf - 45dpf	0,910931
					1	30dpf - 120dpf	0,532521
					1	45dpf - 120dpf	0,127693
					2	7dpf - 21dpf	0,159556
					2	7dpf - 30dpf	0,086664
					2	7dpf - 45dpf	0,549004
Novel Tank Test – Bottom Frequency	RM-ANOVA	(4,54) = 4,386489463	0,003827 134	Tukey	2	7dpf - 120dpf	0,911808
					2	21dpf - 30dpf	0,998362
					2	21dpf - 45dpf	0,931634
					2	21dpf - 120dpf	0,578945
					2	30dpf - 45dpf	0,813547
					2	30dpf - 120dpf	0,399497
					2	45dpf - 120dpf	0,958158
					3	7dpf - 21dpf	0,020934
					3	7dpf - 30dpf	0,009806
					3	7dpf - 45dpf	0,173941
					3	7dpf - 120dpf	0,83208
					3	21dpf - 30dpf	0,998651
					3	21dpf - 45dpf	0,888227
					3	21dpf - 120dpf	0,210891
					3	30dpf - 45dpf	0,752912
					3	30dpf - 120dpf	0,121057
					3	45dpf - 120dpf	0,728203
Novel Tank Test – Bottom Frequency	RM-ANOVA	(4,54) = 4,386489463	0,003827 134	Tukey	4	7dpf - 21dpf	0,010102
					4	7dpf - 30dpf	0,001363
					4	7dpf - 45dpf	0,022754
					4	7dpf - 120dpf	0,616234

					4	21dpf - 30dpf	0,961442
					4	21dpf - 45dpf	0,998204
					4	21dpf - 120dpf	0,259883
					4	30dpf - 45dpf	0,866641
					4	30dpf - 120dpf	0,064592
					4	45dpf - 120dpf	0,41632
					5	7dpf - 21dpf	0,226605
					5	7dpf - 30dpf	0,405293
					5	7dpf - 45dpf	0,086507
					5	7dpf - 120dpf	0,17039
					5	21dpf - 30dpf	0,996093
					5	21dpf - 45dpf	0,988839
					5	21dpf - 120dpf	0,99987
					5	30dpf - 45dpf	0,915045
					5	30dpf - 120dpf	0,985098
					5	45dpf - 120dpf	0,997463
Novel Tank Test – Bottom Time	RM-ANOVA	(4,54) = 4,386489463	0,000310885	Tukey	1	7dpf - 21dpf	0,999926
					1	7dpf - 30dpf	0,966259
					1	7dpf - 45dpf	0,601386
					1	7dpf - 120dpf	0,086709
					1	21dpf - 30dpf	0,929751
					1	21dpf - 45dpf	0,494803
					1	21dpf - 120dpf	0,103224
					1	30dpf - 45dpf	0,924728
					1	30dpf - 120dpf	0,013825
					1	45dpf - 120dpf	0,001173
					2	7dpf - 21dpf	0,999842
					2	7dpf - 30dpf	0,923425
					2	7dpf - 45dpf	0,175064
					2	7dpf - 120dpf	0,458859
					2	21dpf - 30dpf	0,961396
					2	21dpf - 45dpf	0,217582
					2	21dpf - 120dpf	0,342556
					2	30dpf - 45dpf	0,586388
					2	30dpf - 120dpf	0,094926
					2	45dpf - 120dpf	0,001653
					3	7dpf - 21dpf	0,99699
					3	7dpf - 30dpf	0,951143
					3	7dpf - 45dpf	0,023004
					3	7dpf - 120dpf	0,209222
					3	21dpf - 30dpf	0,995133
Novel Tank Test – Bottom Time	RM-ANOVA	(4,54) = 4,386489463	0,000310885	Tukey	3	21dpf - 45dpf	0,047214
					3	21dpf - 120dpf	0,092319
					3	30dpf - 45dpf	0,115586
					3	30dpf - 120dpf	0,0365
					3	45dpf - 120dpf	1,45E-05

					4	7dpf - 21dpf	0,978357
					4	7dpf - 30dpf	0,95983
					4	7dpf - 45dpf	0,275468
					4	7dpf - 120dpf	0,392832
					4	21dpf - 30dpf	0,999967
					4	21dpf - 45dpf	0,587315
					4	21dpf - 120dpf	0,127819
					4	30dpf - 45dpf	0,656169
					4	30dpf - 120dpf	0,101
					4	45dpf - 120dpf	0,002556
					5	7dpf - 21dpf	0,722041
					5	7dpf - 30dpf	0,995807
					5	7dpf - 45dpf	0,997278
					5	7dpf - 120dpf	0,040614
					5	21dpf - 30dpf	0,896258
					5	21dpf - 45dpf	0,492287
					5	21dpf - 120dpf	0,447175
					5	30dpf - 45dpf	0,950918
					5	30dpf - 120dpf	0,086382
					5	45dpf - 120dpf	0,013785
Novel Tank Test – Top Frequency	RM-ANOVA	(4,54) = 2,814510573	0,034054 122	Tukey	1	7dpf - 21dpf	0,136101
					1	7dpf - 30dpf	0,042184
					1	7dpf - 45dpf	0,558533
					1	7dpf - 120dpf	0,999973
					1	21dpf - 30dpf	0,984927
					1	21dpf - 45dpf	0,900201
					1	21dpf - 120dpf	0,096618
					1	30dpf - 45dpf	0,625172
					1	30dpf - 120dpf	0,027189
					1	45dpf - 120dpf	0,471058
					2	7dpf - 21dpf	0,624127
					2	7dpf - 30dpf	0,194035
					2	7dpf - 45dpf	0,879253
					2	7dpf - 120dpf	0,998794
					2	21dpf - 30dpf	0,925961
					2	21dpf - 45dpf	0,988994
					2	21dpf - 120dpf	0,770809
					2	30dpf - 45dpf	0,698288
					2	30dpf - 120dpf	0,291005
					2	45dpf - 120dpf	0,958527
					3	7dpf - 21dpf	0,410341
					3	7dpf - 30dpf	0,043137
Novel Tank Test – Top Frequency	RM-ANOVA	(4,54) = 2,814510573	0,034054 122	Tukey	3	7dpf - 45dpf	0,371037
					3	7dpf - 120dpf	0,97001
					3	21dpf - 30dpf	0,777871
					3	21dpf - 45dpf	0,999994

					3	21dpf - 120dpf	0,777871
					3	30dpf - 45dpf	0,815004
					3	30dpf - 120dpf	0,159146
					3	45dpf - 120dpf	0,738104
					4	7dpf - 21dpf	0,040759
					4	7dpf - 30dpf	0,003922
					4	7dpf - 45dpf	0,236846
					4	7dpf - 120dpf	0,823771
					4	21dpf - 30dpf	0,911799
					4	21dpf - 45dpf	0,92124
					4	21dpf - 120dpf	0,339672
					4	30dpf - 45dpf	0,453632
					4	30dpf - 120dpf	0,061556
					4	45dpf - 120dpf	0,828566
					5	7dpf - 21dpf	0,784971
					5	7dpf - 30dpf	0,359517
					5	7dpf - 45dpf	0,670752
					5	7dpf - 120dpf	0,449977
					5	21dpf - 30dpf	0,950788
					5	21dpf - 45dpf	0,99963
					5	21dpf - 120dpf	0,980122
					5	30dpf - 45dpf	0,984906
					5	30dpf - 120dpf	0,999847
					5	45dpf - 120dpf	0,996284
Novel Tank Test – Top Time	RM-ANOVA	(4,54) = 4,006992148	0,006432 939	Tukey	1	7dpf - 21dpf	0,996685
					1	7dpf - 30dpf	0,996538
					1	7dpf - 45dpf	0,99592
					1	7dpf - 120dpf	0,047518
					1	21dpf - 30dpf	0,951072
					1	21dpf - 45dpf	0,947242
					1	21dpf - 120dpf	0,094914
					1	30dpf - 45dpf	1
					1	30dpf - 120dpf	0,015521
					1	45dpf - 120dpf	0,014859
					2	7dpf - 21dpf	0,99941
					2	7dpf - 30dpf	0,970953
					2	7dpf - 45dpf	0,71362
					2	7dpf - 120dpf	0,193361
					2	21dpf - 30dpf	0,907952
					2	21dpf - 45dpf	0,552113
					2	21dpf - 120dpf	0,26842
					2	30dpf - 45dpf	0,964531
					2	30dpf - 120dpf	0,04216
Novel Tank Test – Top Time	RM-ANOVA	(4,54) = 4,006992148	0,006432 939	Tukey	2	45dpf - 120dpf	0,006979
					3	7dpf - 21dpf	0,998302
					3	7dpf - 30dpf	0,988801

					3	7dpf - 45dpf	0,523788
					3	7dpf - 120dpf	0,180273
					3	21dpf - 30dpf	0,931555
					3	21dpf - 45dpf	0,327213
					3	21dpf - 120dpf	0,284291
					3	30dpf - 45dpf	0,799573
					3	30dpf - 120dpf	0,054899
					3	45dpf - 120dpf	0,002431
					4	7dpf - 21dpf	0,999993
					4	7dpf - 30dpf	0,994803
					4	7dpf - 45dpf	0,985315
					4	7dpf - 120dpf	0,382507
					4	21dpf - 30dpf	0,99744
					4	21dpf - 45dpf	0,990998
					4	21dpf - 120dpf	0,320403
					4	30dpf - 45dpf	0,99994
					4	30dpf - 120dpf	0,178973
					4	45dpf - 120dpf	0,138841
					5	7dpf - 21dpf	0,414528
					5	7dpf - 30dpf	0,9671
					5	7dpf - 45dpf	0,774817
					5	7dpf - 120dpf	0,031982
					5	21dpf - 30dpf	0,790818
					5	21dpf - 45dpf	0,974105
					5	21dpf - 120dpf	0,704476
					5	30dpf - 45dpf	0,984318
					5	30dpf - 120dpf	0,128677
					5	45dpf - 120dpf	0,339014
Novel Tank Test – Immobile Time	RM-ANOVA	(4,54) = 5,692826281	0,000674395	Tukey	1	7dpf - 21dpf	0,04587
					1	7dpf - 30dpf	0,003477
					1	7dpf - 45dpf	0,107491
					1	7dpf - 120dpf	0,966519
					1	21dpf - 30dpf	0,878446
					1	21dpf - 45dpf	0,995766
					1	21dpf - 120dpf	0,006303
					1	30dpf - 45dpf	0,682264
					1	30dpf - 120dpf	0,000313
					1	45dpf - 120dpf	0,018247
					2	7dpf - 21dpf	0,063367
					2	7dpf - 30dpf	0,008149
					2	7dpf - 45dpf	0,091274
					2	7dpf - 120dpf	1
					2	21dpf - 30dpf	0,932937
					2	21dpf - 45dpf	0,999843
Novel Tank Test – Immobile Time	RM-ANOVA	(4,54) = 5,692826281	0,000674395	Tukey	2	21dpf - 120dpf	0,050132
					2	30dpf - 45dpf	0,876013

					2	30dpf - 120dpf	0,005822
					2	45dpf - 120dpf	0,073736
					3	7dpf - 21dpf	0,120526
					3	7dpf - 30dpf	0,015408
					3	7dpf - 45dpf	0,116633
					3	7dpf - 120dpf	0,999994
					3	21dpf - 30dpf	0,913362
					3	21dpf - 45dpf	1
					3	21dpf - 120dpf	0,091136
					3	30dpf - 45dpf	0,918585
					3	30dpf - 120dpf	0,010195
					3	45dpf - 120dpf	0,087969
					4	7dpf - 21dpf	0,062964
					4	7dpf - 30dpf	0,007373
					4	7dpf - 45dpf	0,076494
					4	7dpf - 120dpf	0,891655
					4	21dpf - 30dpf	0,923155
					4	21dpf - 45dpf	0,999988
					4	21dpf - 120dpf	0,352575
					4	30dpf - 45dpf	0,89361
					4	30dpf - 120dpf	0,070702
					4	45dpf - 120dpf	0,400013
					5	7dpf - 21dpf	0,048314
					5	7dpf - 30dpf	0,030364
					5	7dpf - 45dpf	0,093248
					5	7dpf - 120dpf	0,38583
					5	21dpf - 30dpf	0,999717
					5	21dpf - 45dpf	0,998501
					5	21dpf - 120dpf	0,824826
					5	30dpf - 45dpf	0,989498
					5	30dpf - 120dpf	0,722657
					5	45dpf - 120dpf	0,936246

2.3. Supplementary Table 2

Supp. Table 2: Arena sizes used in Developmental Stages

Stage	Arena Size (L x W x H) (cm)
Early Larval (7dpf)	5 x 2 x 3
Late Larval (21dpf)	8 x 3 x 4
Early Juvenile (~30dpf)	10 x 4 x 5

Late Juvenile (~45dpf)	15 x 5 x 8
Adult (<90dpf)	30 x 10 x 15
