# Supplementary material for: Ontogeny of zebrafish behaviors: comparative evaluation of locomotor, social and anxiety parameters in larval, juvenile and adult stages
Source: Lab Anim (NY). 2026 Apr 7;55(5):172–80. doi: 10.1038/s41684-026-01712-x (PMC13143822; doi:10.1038/s41684-026-01712-x)
Supplement: Supplementary file 2 — Reporting Summary [file 41684_2026_1712_MOESM2_ESM.pdf]

nature portfolio

Corresponding author(s): Bonan, C.D

Last updated by author(s): 2025/01/15

Reporting Summary

Nature Portfolio wishes to improve the reproducibility of the work that we publish. This form provides structure for consistency and transparency in reporting. For further information on Nature Portfolio policies, see our Editorial Policies and the Editorial Policy Checklist.

Statistics

For all statistical analyses, confirm that the following items are present in the figure legend, table legend, main text, or Methods section.

n/a	Confirmed	
☐	☒	The exact sample size (*n*) for each experimental group/condition, given as a discrete number and unit of measurement
☐	☒	A statement on whether measurements were taken from distinct samples or whether the same sample was measured repeatedly
☐	☒	The statistical test(s) used AND whether they are one- or two-sided<br>*Only common tests should be described solely by name; describe more complex techniques in the Methods section.*
☐	☒	A description of all covariates tested
☐	☒	A description of any assumptions or corrections, such as tests of normality and adjustment for multiple comparisons
☒	☐	A full description of the statistical parameters including central tendency (e.g. means) or other basic estimates (e.g. regression coefficient) AND variation (e.g. standard deviation) or associated estimates of uncertainty (e.g. confidence intervals)
☐	☒	For null hypothesis testing, the test statistic (e.g. *F*, *t*, *r*) with confidence intervals, effect sizes, degrees of freedom and *P* value noted<br>*Give P values as exact values whenever suitable.*
☒	☐	For Bayesian analysis, information on the choice of priors and Markov chain Monte Carlo settings
☒	☐	For hierarchical and complex designs, identification of the appropriate level for tests and full reporting of outcomes
☒	☐	Estimates of effect sizes (e.g. Cohen's *d*, Pearson's *r*), indicating how they were calculated

Our web collection on statistics for biologists contains articles on many of the points above.

Software and code

Policy information about availability of computer code

Data collection	All videos were obtained during the test and tracking of animals was evaluated using Ethovision XT v.14 software (Noldus, Wageningen, The Netherlands).
Data analysis	After extraction from Ethovision data was analysed in R 4.4.1, using the code present in the files avaiable at https://osf.io/4an63/

For manuscripts utilizing custom algorithms or software that are central to the research but not yet described in published literature, software must be made available to editors and reviewers. We strongly encourage code deposition in a community repository (e.g. GitHub). See the Nature Portfolio guidelines for submitting code & software for further information.

Data

Policy information about availability of data

All manuscripts must include a data availability statement. This statement should provide the following information, where applicable:

- Accession codes, unique identifiers, or web links for publicly available datasets
- A description of any restrictions on data availability
- For clinical datasets or third party data, please ensure that the statement adheres to our policy

The data for all experiments are available at (https://osf.io/4an63/).

Field-specific reporting

Please select the one below that is the best fit for your research. If you are not sure, read the appropriate sections before making your selection.

☒ Life sciences ☐ Behavioural & social sciences ☐ Ecological, evolutionary & environmental sciences

For a reference copy of the document with all sections, see nature.com/documents/nr-reporting-summary-flat.pdf

Life sciences study design

All studies must disclose on these points even when the disclosure is negative.

Sample size	Sample size was based on previous works for the species. 12 fish per life stage were tested
Data exclusions	All tests where the error values "Missing Samples" or "Subject not Found" surpassed 20% in the Ethovision tracking were excluded from analysis. Additionally, data was treated for removal of outliers using the interquartile range (IQR) method
Replication	Each test was conducted in at least two different days, with animals from different breedings and tanks.
Randomization	All animals in the study were of the same group - control animals - thus randomization and blinding was not possible.
Blinding	All animals in the study were of the same group - control animals - thus randomization and blinding was not possible.

Reporting for specific materials, systems and methods

We require information from authors about some types of materials, experimental systems and methods used in many studies. Here, indicate whether each material, system or method listed is relevant to your study. If you are not sure if a list item applies to your research, read the appropriate section before selecting a response.

Materials & experimental systems

n/a	Involved in the study	
☒	☐	Antibodies
☒	☐	Eukaryotic cell lines
☒	☐	Palaeontology and archaeology
☐	☒	Animals and other organisms
☒	☐	Human research participants
☒	☐	Clinical data
☒	☐	Dual use research of concern

Methods

n/a	Involved in the study	
☒	☐	ChIP-seq
☒	☐	Flow cytometry
☒	☐	MRI-based neuroimaging

Animals and other organisms

Policy information about studies involving animals; ARRIVE guidelines recommended for reporting animal research

Laboratory animals	Wild-type AB strain zebrafish at approximate ages of 7, 21, 30, 45 and 120 dpf
Wild animals	*Provide details on animals observed in or captured in the field; report species, sex and age where possible. Describe how animals were caught and transported and what happened to captive animals after the study (if killed, explain why and describe method; if released, say where and when) OR state that the study did not involve wild animals.*
Field-collected samples	*For laboratory work with field-collected samples, describe all relevant parameters such as housing, maintenance, temperature, photoperiod and end-of-experiment protocol OR state that the study did not involve samples collected from the field.*
Ethics oversight	All protocols were approved by the Institutional Animal Care Committee from Pontifícia Universidade Católica do Rio Grande do Sul (CEUA-PUCRS, permit number 10617), and the study followed the guidelines of the National Animal Experimentation Control Council (CONCEA).

Note that full information on the approval of the study protocol must also be provided in the manuscript.
